# Periostin regulates autophagy through integrin α5β1 or α6β4 and an AKT‐dependent pathway in colorectal cancer cell migration

**DOI:** 10.1111/jcmm.15756

**Published:** 2020-09-29

**Authors:** Suyanee Thongchot, Ekapot Singsuksawat, Nuttavut Sumransub, Ananya Pongpaibul, Attaporn Trakarnsanga, Peti Thuwajit, Chanitra Thuwajit

**Affiliations:** ^1^ Department of Immunology Faculty of Medicine Siriraj Hospital Mahidol University Bangkok Thailand; ^2^ Siriraj Center of Research Excellence for Cancer Immunotherapy Faculty of Medicine Siriraj Hospital Mahidol University Bangkok Thailand; ^3^ Department of Pathology Faculty of Medicine Siriraj Hospital Mahidol University Bangkok Thailand; ^4^ Department of Surgery Faculty of Medicine Siriraj Hospital Mahidol University Bangkok Thailand

**Keywords:** autophagy, cancer‐associated fibroblast, colorectal cancer, integrin, periostin

## Abstract

Colorectal cancer (CRC) is one of the most fatal cancers with highly invasive properties. The progression of CRC is determined by the driving force of periostin (PN) from cancer‐associated fibroblasts (CAFs) in the tumour microenvironment. This present work aims to investigate autophagy‐mediated CRC invasion via the receptor integrin (ITG) by PN. The level of PN in 410 clinical CRC tissues was found increased and was an independent poor prognosis marker (HR = 2.578, 95% CI = 1.218‐5.457, *P*‐value = .013) with a significant correlation with overall survival time (*P*‐value < .001). PN activated proliferation, migration and invasion of CRC cells, but with reduced autophagy. Interestingly, the reduction of LC3 autophagic protein corresponded to the increased ability of CRC cell migration. The si*ITGα5*‐treated HT‐29 and si*ITGβ4*‐treated HCT‐116 CRC cells attenuated epithelial‐to‐mesenchymal transitions (EMT)‐related genes and pAKT compared with those in si*ITG*‐untreated cells. The reduction of pAKT by a PI3K inhibitor significantly restored autophagy in CRC cells. These evidences confirmed the effect of PN through either ITGα5β1 or ITGα6β4 and the AKT‐dependent pathway to control autophagy‐regulated cell migration. In conclusion, these results exhibited the impact of PN activation of ITGα5β1 or ITGα6β4 through pAKT in autophagy‐mediated EMT and migration in CRC cells.

## 1 INTRODUCTION

Colorectal cancer (CRC) is the most common gastrointestinal malignancy worldwide with a remarkably high mortality rate.^1^ It is the second leading cause of cancer death and over 50,000 deaths annually are estimated in the United States.^2^ The prognosis of CRC is clearly related to the level of the tumour penetration through the bowel wall and the presence or absence of nodal invasion.^3^ The mechanism of how CRC cells can migrate and metastasize is an active field of study.

Periostin (PN) is a secreted extracellular matrix protein mainly produced by mesenchymal fibroblasts in osteoblasts, periosteum, periodontal ligaments and gastrointestinal tracts.^4^, ^5^ PN is commonly expressed in cancer‐associated fibroblasts (CAFs) of many cancers including oesophageal, gastric, colorectal, prostate, breast and ovarian.^6^, ^7^, ^8^, ^9^, ^10^ In CRC, PN promotes cancer cell survival, angiogenesis and resistance to chemotherapeutic drugs; is also involved in epithelial‐mesenchymal transitions (EMT) leading to cancer invasiveness and metastasis; and is related to poor prognosis in several cancers including colon, pancreatic, ovarian, breast, head and neck, thyroid and gastric.^4^, ^11^, ^12^ The receptor integrin (ITG) has been proven to be activated by PN through the AKT/PKB signalling pathway to promote these tumorigenic properties.^13^

The importance of autophagy is becoming widely recognized as it demonstrates both pro‐ and anti‐tumorigenic functions.^14^ In CRC, cell autonomy and non‐autonomous roles for autophagy are essential in growth and progression; however, the mechanisms downstream of autophagy to reduce or enhance tumour growth are not well known.^15^ It was recently reported that autophagy protein expression levels in mouse osteoblasts were increased after PN silencing.^16^ Up to present knowledge, the alterations of autophagy on CRC tumour promotion after exposure to PN are still unclear.

In this study, 410 CRC patient samples were investigated for PN expression status using immunohistochemistry and the correlation of PN levels to clinicopathological features and disease‐free survivals of the patients were analysed to validate the impact of PN. Additionally, the role of PN in reduction of autophagy in CRC cells through an ITG‐dependent pathway was shown for the regulation of PN‐stimulated CRC cell migration. It is likely to conclude that CAFs‐derived PN affects cancer cells through the ITGα5β1 or the α6β4, via an AKT‐dependent signalling pathway to attenuate autophagy leading to the induction of cancer cell migration. The molecules in the PN‐ITG‐autophagy axis are proposed as targets to attenuate disease progression in CRC patients.

## 2 MATERIALS AND METHODS

### 2.1 Patient samples and culture of CRC cell lines

All 410 formalin‐fixed paraffin‐embedded CRC clinical samples were retrieved from the database of Cancer Registry Unit from 2009 to 2015 and kept at Department of Pathology, Faculty of Medicine Siriraj Hospital, Mahidol University. The sample requests for this study were approved by the Ethics Committee for Human Research of Siriraj Hospital or the Siriraj Institution Review Board (COA no. Si544/2015).

For CRC cell line culture, HCT‐116 cells were maintained in RPMI 1640 medium (Gibco‐BRL, Invitrogen, Carlsbad, CA, USA); HT‐29, SW‐480 and SW‐620 cells were cultured in DMEM medium (Gibco‐BRL), which supplemented with 10% heat‐inactivated foetal bovine serum (FBS) (Gibco‐BRL), 100 U/ml penicillin (Invitrogen, Thermo Fisher Scientific, Inc, Waltham, MA, USA), 100 µg/ml streptomycin (Invitrogen) and amphotericin B (Mediorals Laboratories, Goa, India). All cells were cultured in a humidified 5% CO_2_ incubator at 37˚C. The PI3K inhibitor treatment was performed by 200 nM GDC‐0941 (Pictilisib, Genentech Inc, South San Francisco, CA, USA).

### 2.2 Immunohistochemistry of PN in human CRC tissues

The 4‐µm paraffin‐embedded CRC tissues were processed for PN staining by the protocol as previously reported.^17^, ^18^ The mouse polyclonal anti‐human PN (Shino‐Test Corporation, Japan) at a dilution of 1:100 was used as primary antibody by incubation overnight at 4oC. The mouse Envision + SystemTM horseradish peroxidase (HRP) labelled polymer (K4001, Dako, Carpinteria, CA, USA) was used as the secondary antibody and incubated with the slides for 30 min at room temperature. The signal was developed by 3, 3’‐ diaminobenzidine (DAB) solution (Dako) for 5 min at room temperature and counterstained with Mayer's haematoxylin. The staining samples were observed under light microscopy and scanned by Aperio AT2 Scanscope (High Volume, Digital Whole Slide Scanning) (Leica Biosystems, Division of Leica Microsystem Inc, Buffalo Grove, IL, USA). The scoring values were evaluated by the percentage of positive staining cells (P) and the intensity of the staining signal (I). For P, 0%‐25%, 26%‐50%, 51%‐75% and 76%‐100% were classified as grades 0, 1, 2 and 3. For I, remained unstained, slightly stained, intermediately stained and strongly stained were classified as 0, 1, 2 and 3. The expression scoring was calculated by P x I that covered the total core of 0‐9. Each stained slide was observed by 2 investigators double‐blinded to the clinical data. Patients with a total score of more than 4 (the median value of the overall scores) were grouped as the high PN expression group whereas those with less than or equal to 4 were in low PN group.

### 2.3 Small interference RNA (siRNA) knockdown of ITGα5 and ITGβ4

Human ITGα5 and ITGβ4 post‐transcriptional silencing in CRC cells was achieved by siRNA: ITGα5 siRNA(h): sc‐29372 (Santa Cruz Biotechnology, Inc, Dallas, TX, USA) for ITGα5; ITGβ4 siRNA (h): sc‐35678 (Santa Cruz Biotechnology, Inc) for ITGβ4. The protocols were followed the manufacturer's instructions. Briefly, 60 pmol siRNA was mixed with 100 µl siRNA transfection medium (Lipofectamine 2000, Invitrogen) to treat 2 x 10^5^ cells. Cells were incubated in the transfection reagent at 37°C in a humidified incubator with 5% CO_2_ for 6 h. After 6 h, the cells were incubated for 48 h in fresh 10% serum containing medium prior to starting the treatment with 100 ng/ml of recombinant PN (rPN) (Biovendor, Heidelberg, Germany).^19^ The fluorescein conjugate‐A (sc‐36869, Santa Cruz Biotechnology, Inc) and the control siRNA‐A (sc‐37007, Santa Cruz Biotechnology, Inc) were utilized as the positive and negative controls.

### 2.4 Real‐time PCR

Total RNA was extracted by the Total RNA Purification Kit (GeneMark, GM Biolab, Taiwan) following the instruction manual. The extracted total RNA was converted to cDNA by MMLV reverse transcriptase using the SuperScript™ III First‐Strand Synthesis System (Invitrogen) following the manufacturer's instructions. The semi‐quantitative expression level of gene of interest was determined by SYBR Green‐based real‐time PCR (Roche Applied Science, Mannheim, Germany). The expression of genes of interest in CRC cells treated with rPN was represented by the crossing point (Cp) compared with that of untreated control cells after normalizing with the Cp of the internal control gene, GAPDH.

### 2.5 Western blotting analyses

Protein samples of SDS‐PAGE were loaded and transferred to a PVDF membrane. The immunodetection was performed with 1:500 mouse anti‐human ITGα5 (sc‐376199, Santa Cruz Biotechnology, Inc), 1:5,000 rabbit anti‐human LC3 (L7543, Sigma‐Aldrich, St. Louis, MO, USA), 1:500 anti‐human p‐AKT (Ser473) (9271, Cell Signaling Technology Inc, Beverly, MA, USA), 1:500 rabbit anti‐human p44/42 MARK (ERK1/2) (9106, Cell Signaling Technology Inc), 1:500 rabbit anti‐human AKT (9272, Cell Signaling Technology Inc), 1:500 rabbit anti‐human p44/42 MARK (ERK1/2) (9102, Cell Signaling Technology Inc), 1:200 mouse anti‐human E‐cad (13‐1700, Thermo Fisher Scientific), 1:500 mouse anti‐human α‐SMA (clone 1A4, Sigma‐Aldrich), 1:500 mouse anti‐human vimentin (sc‐6260, Santa Cruz Biotechnology, Inc), and 1:500 mouse anti‐human TWIST‐2 (ab57997, Abcam, UK) and 1:10,000 mouse anti‐human β‐actin (ACTB) (sc‐47778, Santa Cruz Biotechnology) for primary antibodies, and incubated with suitable secondary antibodies under suitable conditions including 1:2,000 HRP‐conjugated goat anti‐mouse IgG antibody (Zymed, Thermo Fisher Scientific, Waltham, MA, USA) for mouse primary antibody, and 1:2,000 HRP‐conjugated goat anti‐rabbit IgG antibody (Abcam) for rabbit primary antibody. The immunoreactive signals were visualized by Enhanced Chemiluminescence Plus solution (ECL) (Perkin Elmer, Waltham, MA, USA) under the Gel Document (Syngene®, Ste. Q Frederick, MD, USA).

### 2.6 Immunofluorescence staining

CRC cells were plated on sterile glass coverslips at a density of 5 x 104 cells and incubated for 24 h in the treatment conditions. At the end, the cells were fixed in ice‐cold methanol and then incubated overnight at 4°C in a humidified chamber with the indicated primary antibodies against PN (1:100) (sc‐49480, Santa Cruz Biotechnology, Inc), ITGα5 (1:100) (sc‐376199, Santa Cruz Biotechnology, Inc), ITGβ4 (1:100) (sc‐14426, Santa Cruz Biotechnology, Inc) and LC3 (1:100) (L7543, Sigma‐Aldrich). After washing out the excess primary antibody, the coverslip was incubated for 3 h at room temperature with the appropriate secondary antibodies: 1:2,000 goat anti‐mouse IgG‐Cy3 (Jackson ImmunoResearch Laboratories Inc, West Grove, PA, USA), 1:1,000 rabbit anti‐mouse IgG‐FITC (Dako) and 1:1,000 donkey anti‐goat IgG Alexa Fluor 594 (Abcam). Hoechst 33 342 solution (Invitrogen) was added to stain the nucleus. Fluorescence was captured with the LSM 800 confocal laser scanning microscope (Carl Zeiss, Jena, Germany). Numbers of PN‐positive cells in the conditions of with and without rPN treatment and numbers of LC3 dots in either PN‐positive or PN‐negative cells were counted in 5 fields (original magnification of 63x).

### 2.7 Three‐dimensional (3D) spheroid formation

Tumour spheroids were initiated by seeding 1x10^3^ CRC cells in a volume of 200 µl into individual wells of pre‐cooled 96‐well ultra‐low attachment plates (#7007, Costar®, Corning, NY, USA). The cell suspensions were supplemented with 2.5% cold Matrigel™ (#354234, BD Biosciences, San Jose, CA, USA) in the complete media. Spheroids were allowed to be established for 96 h at 37ºC in a humidified incubator with 5% CO2. After 96 h, 100 ng/µl recombinant PN (rPN) (Biovendor, Candler, NC, USA) was added, imaging was taken for determining tumour spheroid growth kinetics at days 4, 7, 10, 13 and 15 after tumour spheroid initiation (day 0). During tumour spheroid progression, invadopodia ^20^ of 3D cell invasion was monitored at 0, 24, 48 and 72 h. Images and data analyses of tumour spheroids were carried out by the CellSens Standard program on an inverted microscope model IX71 (Olympus, Waltham, MA, USA). The mean radius was used to calculate the volume with the formula 4/3πr3.

### 2.8 Cell migration and invasion assay

CRC cells were cultured in 24‐well plates until more than 90% of confluence was reached, scratched with a yellow tip and then incubated in 100 ng/ml rPN (Biovendor) and the wound area was visualized and digitally photographed under an inverted microscope (IX71/IX51, Olympus) at 0, 24, 48 and 72 h. Quantification of cell migration was determined with the formula: % Migration area = [(Area of original wound ‐ Area of wound after healing)/ Area of original wound] × 100.

Moreover, the transwell cell migration assay was performed in a modified 24‐well Boyden chamber (Costar®) with a polycarbonate membrane of 8.0 μm pores. The 50,000 cells/well were seeded in the upper chamber insert and incubated in 1% FBS DMEM (for HT‐29) or RPMI 1640 (for HCT‐116) medium in the presence or absence of rPN (Biovendor), as indicated. Completed DMEM (for HT‐29) or RPMI 1640 (for HCT‐116) medium was added to the lower chamber. After 24, 48 and 72 h, cells that passed the membrane were fixed with methanol, stained with 0.5% crystal violet, and the migrated cells quantitated with ImageJ software version 1.52a.

### 2.9 Statistical analysis

The correlation of PN expression in CRC tissues with clinicopathological data was verified by Fisher's exact univariate analysis. The Kaplan‐Meier log rank test was used for survival analysis. The Cox proportional hazards model was used to examine the association of each clinicopathological parameter with overall survival. The significant parameters were further determined in stepwise modelled multivariate analysis. The *P*‐value of less than .05 was considered statistically significant. All statistical analyses were performed with SPSS version 23 (SPSS Inc, Chicago, IL, USA).

## 3 RESULTS

### 3.1 Demographics and clinicopathological parameters correlation

**Figure 1:**
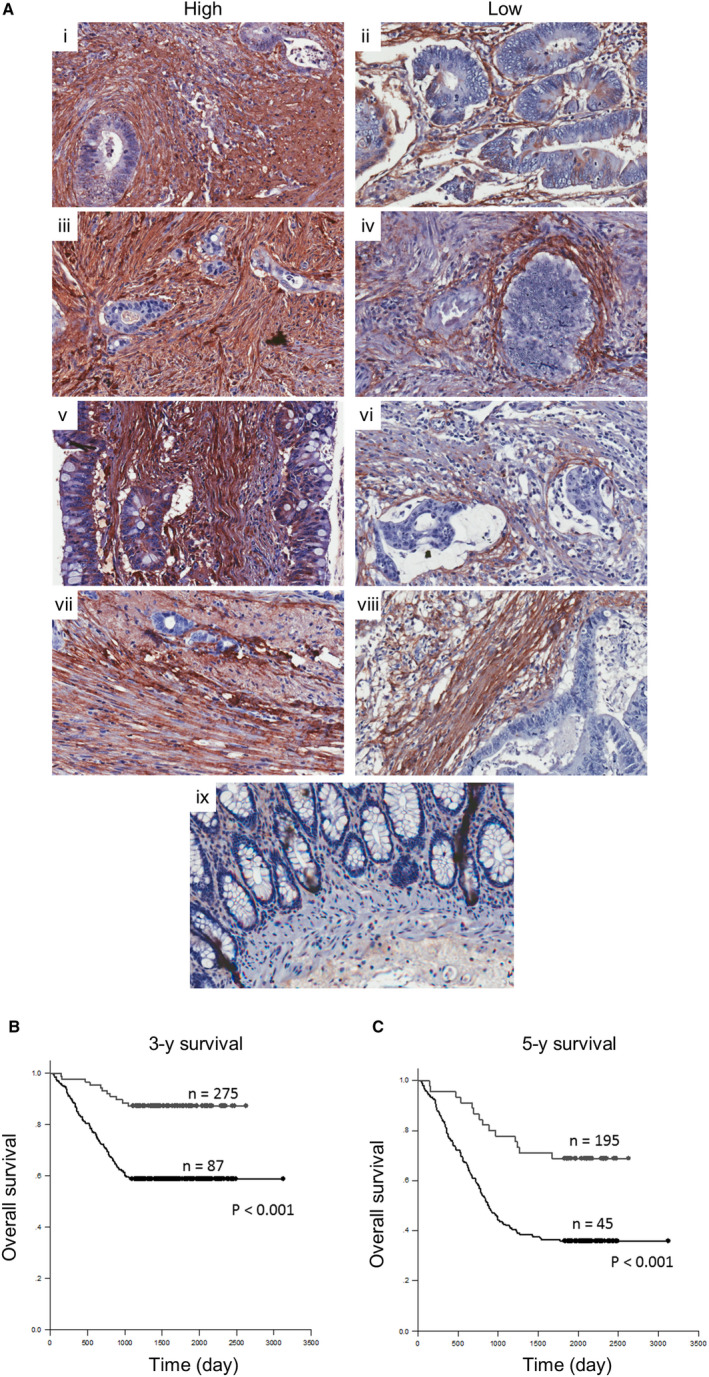
Immunohistochemical staining of PN in CRC tissues. A, Representative pictures of high and low levels of PN at different CRC stages: (i and ii) stage IV, (iii and iv) stage III, (v and vi) stage II and (vii and viii) stage I; and (ix) shows normal colorectal tissue. Original magnification of 200x. Kaplan‐Meier log rank for survival analysis using (B) 3‐years and (C) 5‐years as a cut‐off value to be evaluated

Among 410 total cases, 232 (57%) cases were male. The patients were aged ranging from 29 to 95 years with a mean age of 64 years (Table 1). The maximal percentagec of TNM staging cases was stage 3 (38%, 156/410), whereas stage 1, 2 and 4 were 12%, 21% and 29%. Serum CEA was monitored in almost all of the cases (n = 401) and showed the correlations with PN levels together with tumour sizes, lymphovascular invasion, perineural invasion, tumour staging, lymph node metastasis and distant metastasis with statistical significance (*P*‐value < .001) (Table 1). PN was predominantly detected in intratumoral fibroblasts (Figure 1A, i‐viii) and was found very low in normal colorectal tissues (Figure 1A, ix). High PN levels were correlated with statistical significance to lymphovascular invasion, perineural invasion, tumour staging, lymph node metastasis and distant metastasis (*P*‐value < .001). Multivariate analysis exhibited the significance of high PN levels in the tissues as an independent risk factor with an HR of 2.578 (*P*‐value = .013). In contrast, serum CEA showed no significant risk factors (Table 2). The lymphovascular (*P*‐value = .002), perineural (*P*‐value = .029) invasions and late stages of tumours (stages III‐IV) (*P*‐value < .001) were independently bad prognosis markers with an HR of more than 1. In addition, 3‐years and 5‐years overall survival using the Kaplan‐Meier log rank test showed high levels of PN expression significantly correlated with patient short survival time with statistical significance (Figure 1B). Interestingly, the level of tissue PN and serum CEA exhibited the significant linear correlation (Table S1).

**Table 1:** Correlation between PN levels with the clinicopathological parameters

Characteristics	PN			Characteristics	CEA		
	Low 0‐3	High 4‐9	*P*‐value		Low	High	*P*‐value
Sex (n = 410)				(n = 401)			
F (n = 178)	49	129	.165	F (n = 174)	66	108	.005
M (n = 232)	50	182		M (n = 227)	56	171	
Age (n = 410)				(n = 401)			
<64 years (n = 200)	50	150	.730	<64 years (n = 196)	63	133	.515
>64 years (n = 210)	49	161		>64 years (n = 205)	59	146	
Tumour size (n = 410)				(n = 401)			
<5 cm (n = 188)	45	143	1.000	<5 cm (181)	68	113	.006^*^
>5 cm (n = 222)	54	168		>5 cm (220)	54	166	
Lymphovascular invasion (n = 402)				(n = 393)			
Absence (n = 240)	78	162	<.001^*^	Absence (n = 234)	90	144	<.001^*^
Presence (n = 162)	20	142		Presence (n = 159)	31	128	
Perineural invasion (n = 396)				(n = 387)			
Absence (n = 233)	72	161	<.001^*^	Absence (n = 227)	88	139	<.001^*^
Presence (n = 163)	24	139		Presence (n = 160)	29	131	
Staging I‐III (n = 410)				(n = 401)			
I‐III (n = 294)	89	205	<.001^*^	I‐III (n = 285)	112	173	<.001^*^
IV (n = 116)	10	106		IV (n = 116)	10	106	
Staging I‐II (n = 410)				(n = 401)			
I‐II (n = 138)	56	82	<.001^*^	I‐II (n = 135)	65	70	<.001^*^
III‐IV (n = 272)	43	229		III‐IV (n = 266)	57	209	
LN metastasis (n = 410)				(n = 401)			
Absence (n = 164)	61	103	<.001^*^	Absence (n = 160)	69	91	<.001^*^
Presence (n = 246)	38	208		Presence (n = 241)	53	188	
Distant metastasis (n = 381)				(n = 373)			
Absence (n = 266)	89	177	<.001^*^	Absence (n = 258)	109	149	<.001^*^
Presence (n = 115)	8	107		Presence (n = 115)	9	106	
Serum CEA (n = 400)							
<3.4 ng/mL (n = 122)	38	84	.031^*^				
>3.4 ng/mL (n = 278)	58	220					

^*^ Represents statistical significance *P*‐value < .05

**Table 2:** Cox's regression multivariate analysis of PN expression and clinicopathological parameters

Characteristics	HR	95% CI	*P*‐value
PN expression in CRC tissues (High)	2.578	1.218‐5.457	.013^*^
Age (>64 years)	0.741	0.458‐1.198	.221
Tumour size (>5 cm)	1.332	0.825‐2.150	.241
Lymphovascular invasion	2.183	1.317‐3.619	.002^*^
Perineural invasion	1.743	1.058‐2.872	.029^*^
Chemotherapeutic treatment	0.314	0.171‐0.578	<.001^*^
Radiation treatment	0.562	0.263‐1.202	.138
Serum CEA (> 3.4 ng/mL)	1.387	0.727‐2.644	.321
Staging III‐IV	9.576	3.741‐24.508	<.001^*^

* Represents statistical significance *P*‐value < .05

### 3.2 PN stimulates CRC spheroid formation and migration

**Figure 2:**
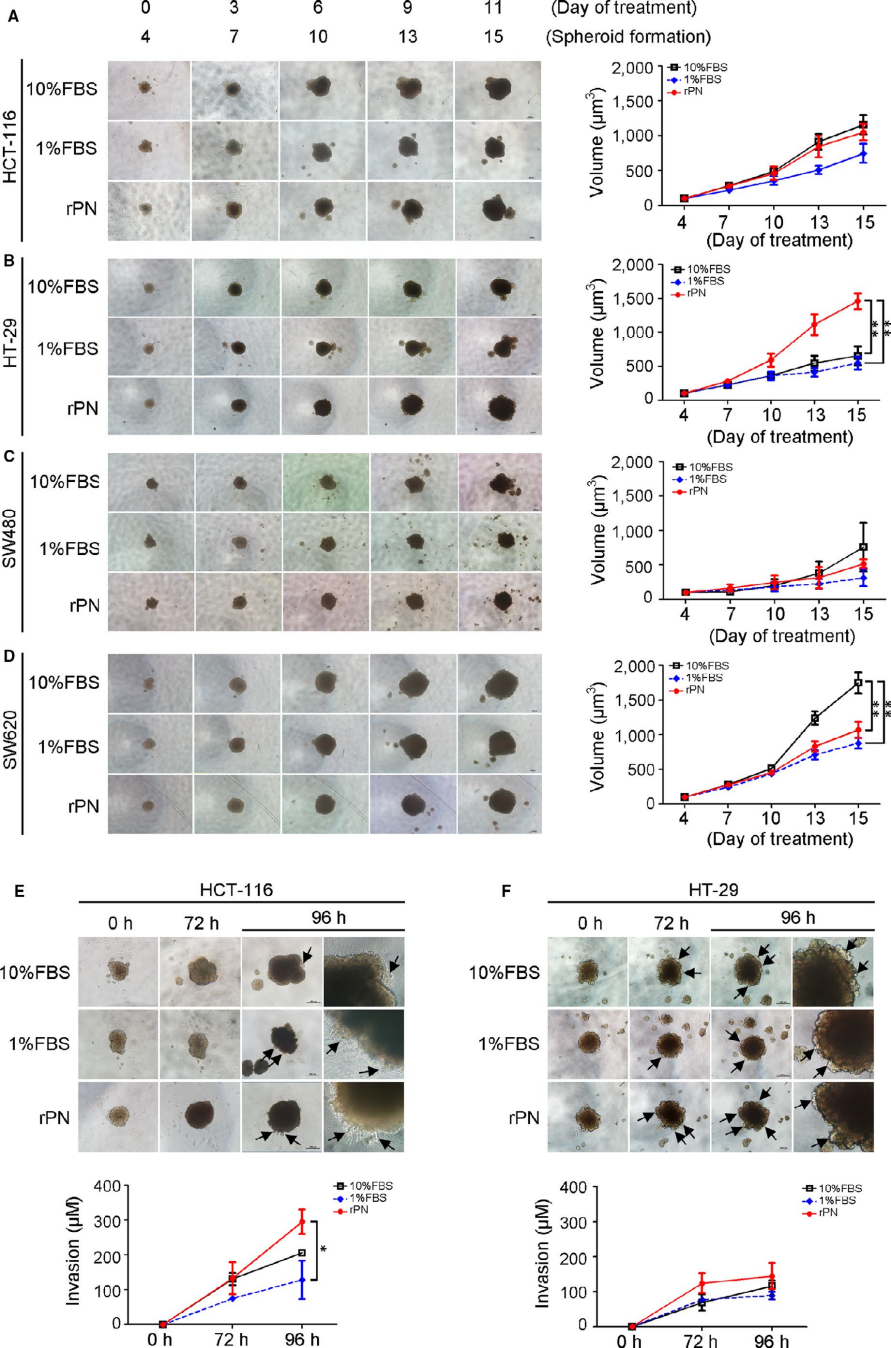
PN induces CRC cell growth and invasion in 3D‐spheroid assay. A‐D, Light microscopy images of CRC spheroid growth in treatment of rPN (100 ng/mL) at day 4, 7, 10, 13 and 15 compared with CRC cells in 10% FBS or 1% FBS media. Spheroid sizes at each time point were calculated and are plotted in volumes (µm^3^). Statistical significance is based on the results of Student's *t* test. * *P*‐value < .05; ** *P*‐value < .01. E, HCT‐116 and (F) HT‐29 spheroid cells moved into Matrigel was observed in rPN treatment. Scale bar = 200 µM; Original magnification of 200x

**Figure 3:**
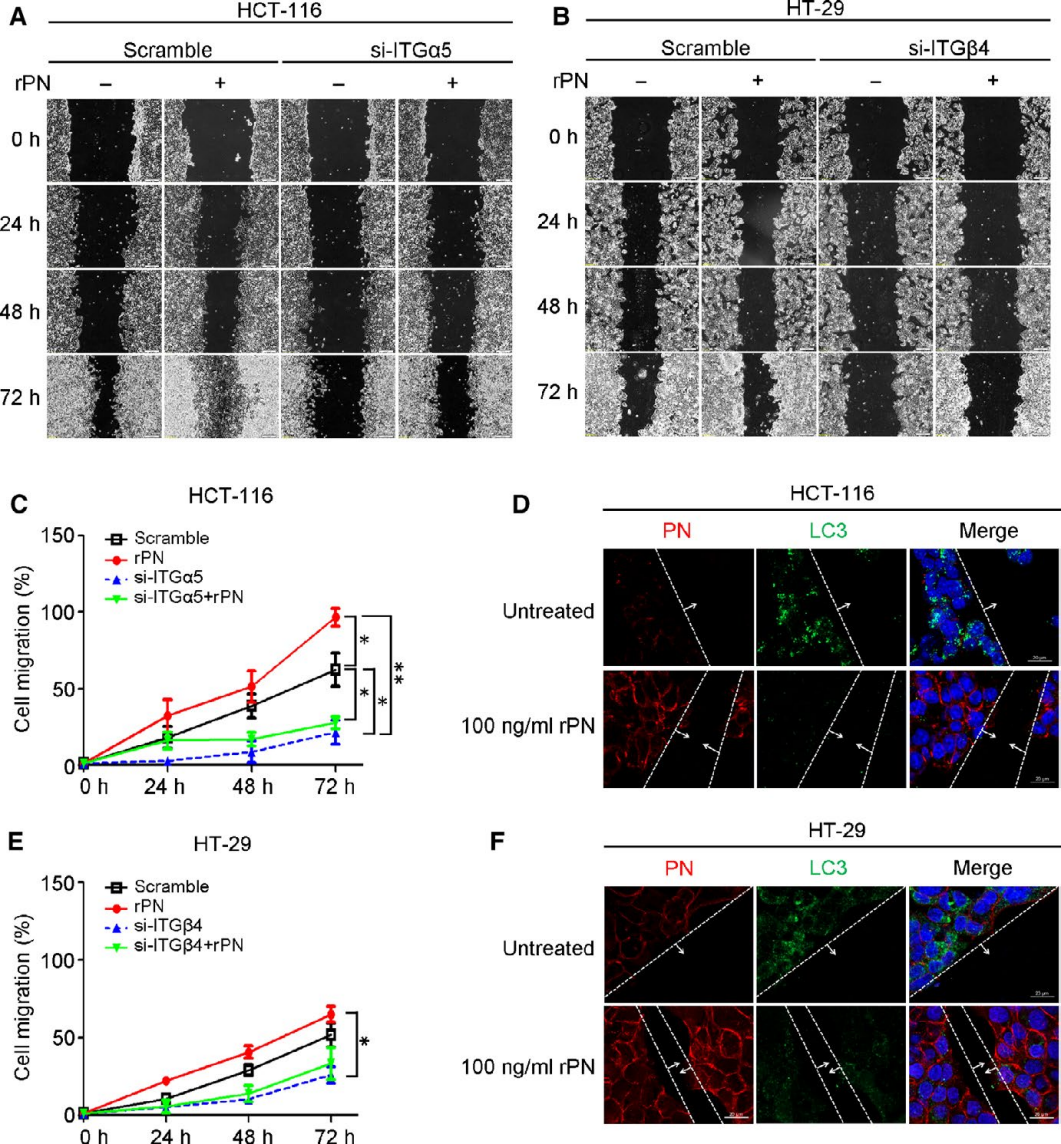
PN promotes CRC cell migration through ITGα5 and ITGβ4. A‐B, Migration ability of HCT‐116 cells transfected with or without si*ITGα5* and HT‐29 cells transfected with or without si*ITGβ4* under rPN treatment were identified by wound‐healing migration assays. C‐D, Percentages of migrated cells are at each time point after PN treatment in CRC cells treated with or without si*ITGα5* or si*ITGβ4*. Bar graph represents mean ± SD; *P‐value < .05; **P‐value < .01 compared between conditioning of treatments. E‐F, Representatives of the expression of LC3 and PN at the migration front in the wound area. Scale bar = 20 µM; Original magnification of 63x

The size of CRC spheroids of all CRC cell lines was increased in a time‐dependent manner when exposed to rPN more than that of negative controls treated with 1% FBS media (Figure 2A‐D), but only HT‐29 showed a significant increase (*P*‐value < .05) (Figure 2B). Interestingly, both HCT‐116 and HT‐29 CRC cells revealed the increased cell invasion that protruded out of the spheroid masses representing the capability of cancer cell invasion (Figure 2E,F) with statistical significance detected in HCT‐116 CRC cells (Figure 2E). In the wound‐healing cell migration assay, rPN induced both CRC cells to move in a 2D way (Figure 3A‐D) but the significant effect was only observed in HCT‐116 CRC cells (Figure 3A,C).

### 3.3 PN reduces migration capability of CRC cells through ITGα5β1 and α6β4

The expression of ITGs on CRC cells was explored and the results revealed high levels of ITGs α5‐ and β1‐subunits in HCT‐116 whereas HT‐29 had high levels of ITGsα6‐ and β4‐subunits (Figure S1). According to the specific pairing of the ITGα5‐subunit to only the ITGβ1‐subunit,^21^ the level of ITGα5‐subunit can imply the level of ITGα5β1. In a similar way, ITGβ4‐subunit binds only to the ITGα6 subunit, so that the ITGβ4‐subunit level represents ITGα6β4 in the cells. The results indicated that HCT‐116 and HT‐29 predominantly had ITGα5β1 and ITGα6β4. Using siITGα5 and siITGβ4, the ITGα5β1‐knockdown HCT‐116 cells and ITGα6β4‐knockdown HT‐29 cells were successfully produced (Figure S2). The migration induction effect of rPN was attenuated in these two ITG‐knockdown cells, HCT‐116 (Figure 3A,C) and HT‐29 cells (Figure 3B,D) with statistical significance compared with control cells.

### 3.4 PN attenuated autophagy in CRC underwent EMT through ITGα5β1 or ITGα6β4 and AKT‐ or ERK‐dependent pathways

**Figure 4:**
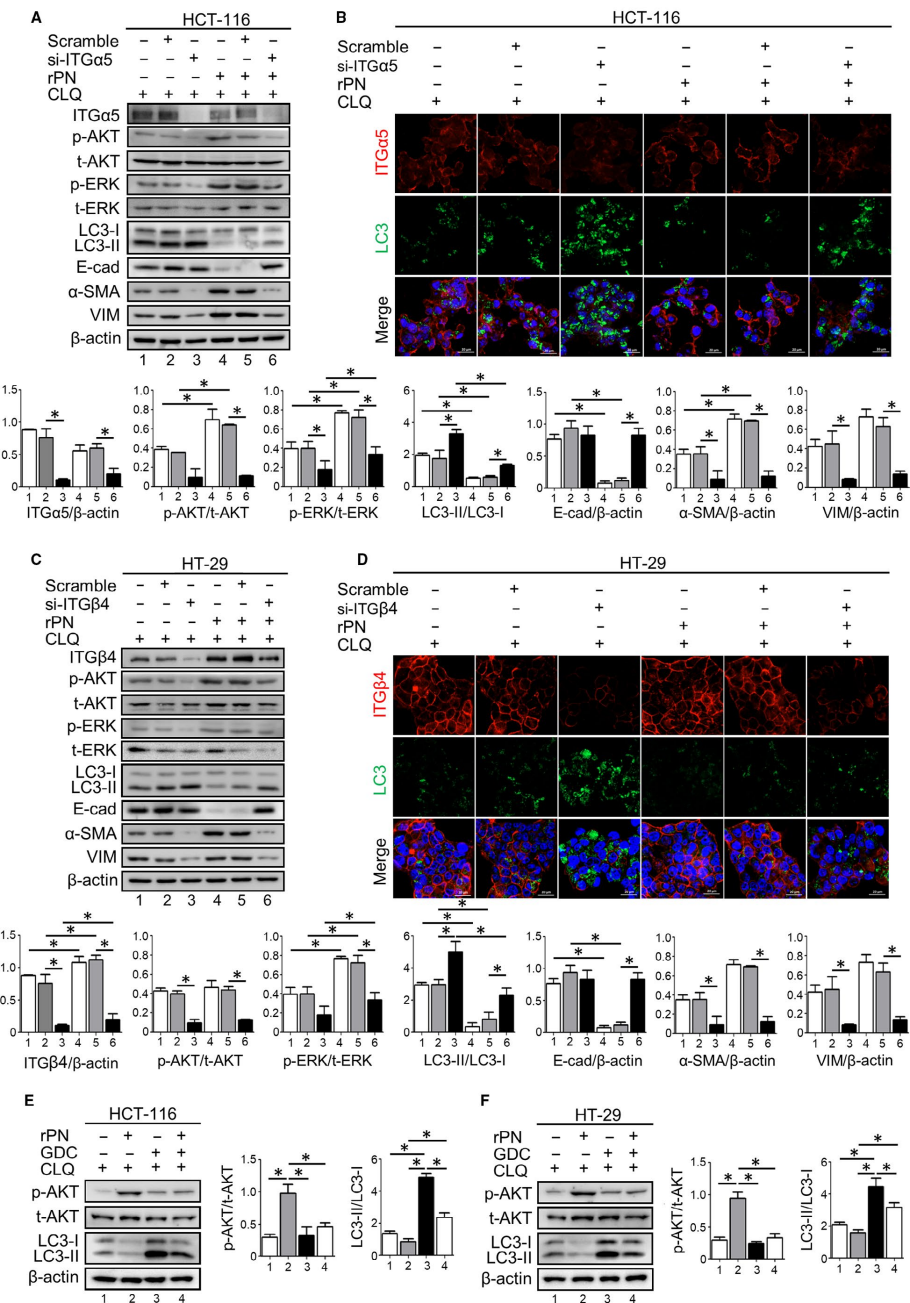
PN promotes CRC cell migration through reduction of autophagy. Expressions of proteins that involved autophagy and EMT pathways were demonstrated by Western blotting and immunofluorescence. A and C, Western blot analyses show the levels of expressions of ITGα5 (A), ITGβ4 (C), pAKT, pERK, LC3, E‐cad, α‐SMA and VIM in HCT‐116 (A) and HT‐29 (C) cells upon incubation for 24 h in rPN. Densitometry of the bands is reported as the ratios of each protein versus β‐actin internal control. Bar graphs represent mean ± SD of three independent experiments. B and D, the marker of autophagy (LC3, green colour) and ITGα5 or ITGβ4 (red colour) were assessed by immunofluorescence in HCT‐116 or HT‐29. The nuclei were counterstained with DAPI. Scale bar = 20 µM; Original magnification of 63x. *P‐value < .05

The expression of LC3 autophagic protein in migratory HCT‐116 and HT‐29 cancer cells at the invasive front of the wound was reduced in rPN‐treated cells as compared to untreated cells in correlation with the increased capability of cancer cell migration (Figure 3E,F). The attenuation of autophagy by rPN was restored in cells having deficient levels of ITGs (Figure 4A,B; Figure 4C,D).

**Figure 5:**
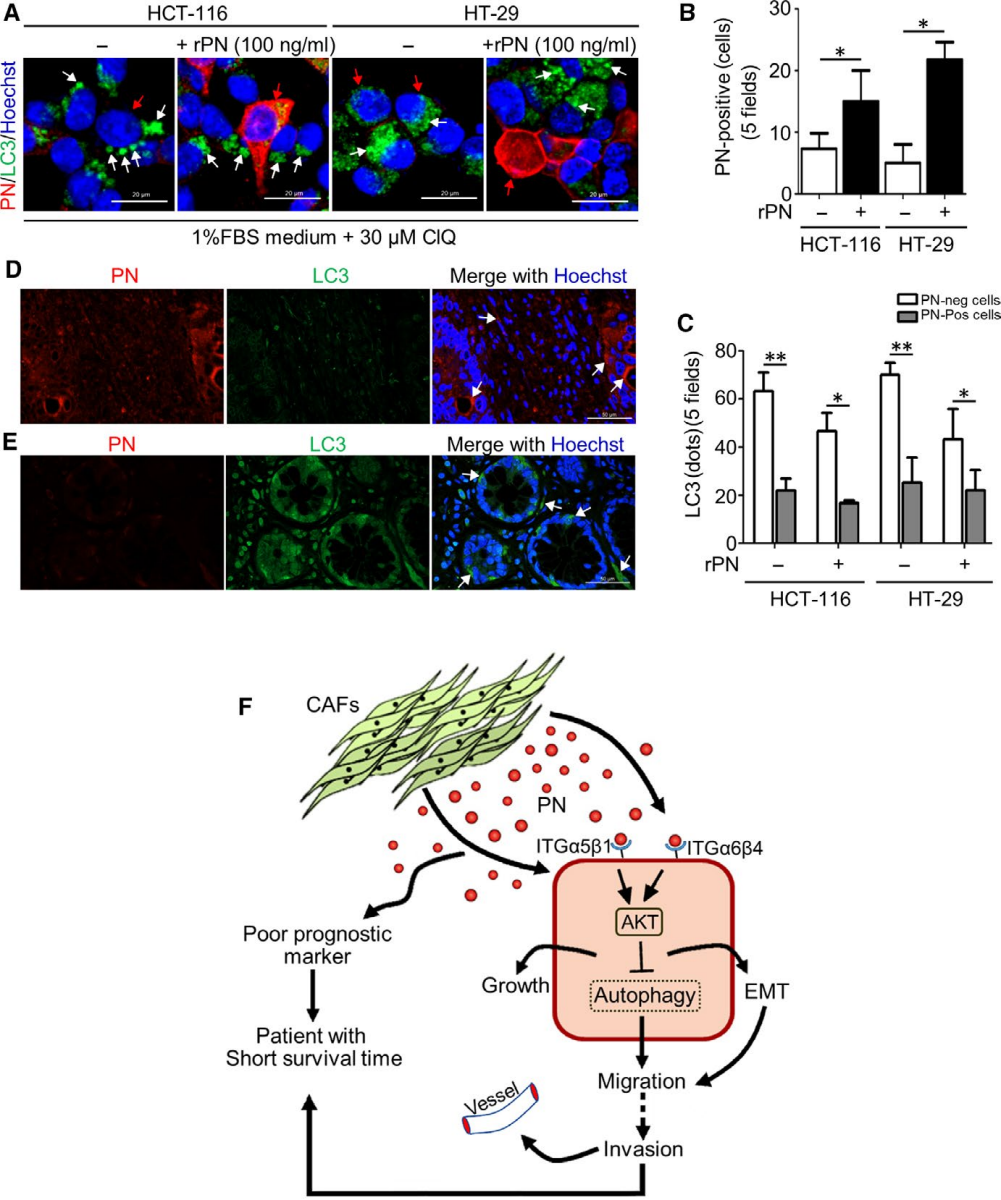
PN and autophagy in CRC cell lines. (A‐C) CRC cells, HCT‐116 and HT‐29 were treated with 100 ng/ml of PN in 1% FBS medium, 30 µM of CLQ was added at 8 h before harvest, and then subjected for IF staining of PN (orange colour) and LC3 (green colour). Bar graph represents mean ± SD; *P‐value < .05; **P‐value < .01. D, E, Co‐localization of PN and LC3 in tumour clinical sections of CRC patients. Scale bar = 20 µM; magnification = 63×. F Schematic diagram illustrates the potential of PN secreted from cancer‐associated fibroblasts (CAFs) to reduce autophagy in CRC‐mediated migration through ITGα5β1 or ITGα6β4 and AKT‐dependent pathways

The rPN significantly induced pAKT and pERK in HCT‐116 CRC cells, but in siITGα5‐treated cells this induction effect of pAKT was attenuated nearly completely (Figure 4A). In contrast, pERK in siITGα5‐treated HCT‐116 cells was still increased by rPN activation similar to scramble‐treated cells. HCT‐116 cells treated with rPN showed significant reductions of E‐cad which is a hallmark of cells that underwent EMT; however, the expression was restored when cells knocked down the expression of ITGα5. The expression of mesenchymal genes including vimentin (VIM) and α‐smooth muscle actin (α‐SMA) revealed up‐regulation after HCT‐116 cells were exposed to rPN, and this increased effect was not detected in siITGα5‐treated HCT‐116 cells. Interestingly, LC3 was significantly reduced in rPN‐treated cells but no alteration of LC3 occurred when cells had a transient defect in ITGα5 expression (Figure 4A,B). Corresponding to these alterations, rPN‐stimulated HCT‐116 cell migration occurred and this effect disappeared in siITGα5‐treated cells (Figure S3). It may be more easily explained in that the expression of the E‐cad epithelial marker was reduced in rPN‐treated cells, whereas the expressions of α‐SMA and VIM mesenchymal markers were increased in CRC cells exposed to rPN in concordance to low level autophagy. Moreover, PN‐regulated pAKT in cells with intact and deficient ITGa5 expression, but not pERK, showed as expected. The same pattern of alterations of EMT genes, pAKT, pERK and LC3 were detected in HT‐29 CRC cells (Figure 4C,D). The phosphatidylinositol‐4,5‐bisphosphate 3‐kinase class 1 (PI3KC1) inhibitor named GDC‐0941 (200 nM) was treated in combination with 100 ng/ml rPN for 18 h. The Western blot results showed that GDC‐0941 dramatically decreased pAKT while increased the ratio of LC3‐II/LC3‐I in both CRC cell lines (Figure 4E,4F). Noteworthy, GDC‐0941 greatly counteracted the autophagy‐negative regulation effect of rPN. GDC‐0941 showed an increased LC3‐II/I ratio of both cells upon rPN treatment when compared with rPN treatment alone (Figure 4E,F). Additionally, the co‐staining of PN and LC3 was performed and exhibited that PN‐treated cells had low expression of LC3 autophagy protein in both HT‐29 and HCT‐116 cells (Figure 5A‐C). Interestingly, CRC tissues with high levels of PN had low levels of LC3 (Figure 5D,E).

## 4 DISCUSSION

PN is well accepted for its up‐regulated expression in many cancers including CRC.^5^, ^8^, ^9^, ^10^ Herein, the increased PN was confirmed in Thai CRC tissues and strongly correlated with patient short survival time as reported previously.^10^, ^13^, ^22^ Moreover, these results supported the findings that CAFs were the major sources of PN production in CRC tissues and CAFs‐derived PN was a poor prognostic marker in CRC patients.^9^, ^10^, ^23^ The mechanism of PN to activate predominant ITGα5β1 or ITGα6β4 receptors on CRC cells to activate cancer cell aggressiveness was revealed.

The association of ITGα1β1 and the progression of CRC has been reported.^24^ The ITGα8 mRNA was reported as a potential diagnostic biomarker whereas ITGα5 may serve as an independent prognosis indicator for CRC.^25^ In the CRC cells described herein, the predominant expressions were ITGα5‐subunit in HCT‐116 and ITGβ4‐subunit in HT‐29 cells. According to the concept of ITG subunits pairing between α‐ and β‐subunits, α5 can bind solely to β1‐subunit^21^; hence, the ITGα5‐subunit level could be utilized to represent the ITGα5β1 level in CRC cells. For α‐subunit ITGs, β4 can bind solely to α6‐subunit,^21^ so that it can be proposed that ITGβ4 can determine ITGα6β4 levels. PN activation on cancer cells have been well accepted to be through ITGs αvβ3, αvβ5, α5β1 and α6β4.^5^, ^19^ In the present study, ITGα5β1 and ITGα6β4 were presented predominantly on CRC cells and could be activated by PN to induce aggressive phenotypes of cancer cells and the inhibition of these two ITGs affected PN’s function. These ensure the impact of PN through ITGα5β1 or ITGα6β4 in CRC cells.

The tumorigenic activities of PN are activated by binding to ITG and activating AKT/PKB‐mediated or FAK‐mediated signalling pathways leading to the increased cell survival, angiogenesis, invasion, metastasis and EMT of cancer cells.^11^ In this present work, induction of tumorigenesis characterized by EMT‐mediated cell invasion through ITGα5β1 or ITGα6β4 was a novel pathway for PN function in CRC cells that was found in addition to the previous report via the ITGαvβ3‐AKT/PKB‐dependent pathway.^13^ The results were confirmed by the increased levels of pAKT and pERK after PN treatment, but not in cells with impaired levels of these two receptors. Notably, the increased level of pERK by PN activation was not attenuated which may have implied no involvement of the ERK pathway. PN‐activated PI3K/AKT/survivin was related to chemoresistance in CRC.^26^ The PN‐ITG‐AKT‐ERK‐dependent pathway is related in cell proliferation and induction of invasion.^19^, ^27^, ^28^ The evidence in this study highlights the roles of PN‐ITGα5β1or PN‐ITGα6β4 through an AKT‐dependent pathway in CRC cell migration.

Importantly, the reduction of autophagy through ITGα5β1 and ITGα6β4 in PN‐driven CRC cell migration was exhibited together with an increment of pAKT, but not pERK. These data could support the findings of PN‐reduced autophagy which was controlled by the activation of the ITGα5β1/AKT signalling pathway. Restoration of autophagy in cancer cells could attenuate EMT and halt cancer cell migration.^29^, ^30^ All the above‐mentioned evidence supported the present findings in CRC cells in which a decreased level of LC3, an autophagic protein was found after exposure to PN, especially in cancer cells at the invasive front of migration. Additionally, EMT‐related proteins including E‐cadherin (E‐cad), α‐SMA and VIM were altered and expressions regulated through ITGα5β1‐ or α6β4‐AKT‐dependent pathways in these highly migrated CRC cells. The ITG‐PI3K‐AKT‐dependent pathway and autophagy reduction has been reported in breast cancer cell migration induced by osteopontin.^31^ In this respect, we are firstly showing that inhibition of AKT counteracts the reduction of autophagy under PN exposure by restoring LC3‐II in CRC cells, confirming PN‐ITG‐AKT‐autophagy signalling‐mediated CRC cell migration. Overall, PN‐reduced autophagy through ITGα5β1 or α6β4‐AKT‐dependent signalling pathways leading to the activation of EMT and migration of CRC cells (Figure 5F). These findings would suggest targeting autophagy as well as PN‐activated signalling pathways as the therapeutic approach to attenuating CRC progression. Additionally, PN is supposed to be a promising poorly prognosis molecule suggesting to clinicians the aggressive treatment in CRC patients with its high levels. Targeting autophagy as well as PN‐ITG‐AKT‐dependent signalling pathway are proposed as the combined therapeutic targets to attenuate CRC progression.

## CONFLICT OF INTEREST

The authors confirm that there are no conflicts of interest.

## AUTHORS’ CONTRIBUTIONS

ST: Experiments, preparation of figures and tables, and manuscript drafting and editing; ES: Experiments and preparation of some figures and tables; NS and AP: Clinical data collection; AT: Clinical samples and the clinical data analysis; PT: Experiments and clinicopathological correlation analysis; CT: Research grant, experiments, data analysis and manuscript drafting, editing and submission.

## Supporting information

Fig S1

Fig S2

Fig S3

Table S1

## Data Availability

Data available on request from the authors.
