# Supplementary figures and images for: Periostin regulates autophagy through integrin α5β1 or α6β4 and an AKT‐dependent pathway in colorectal cancer cell migration
Source: J Cell Mol Med. 2020 Sep 29;24(21):12421–32. doi: 10.1111/jcmm.15756 (PMC7686974; doi:10.1111/jcmm.15756)

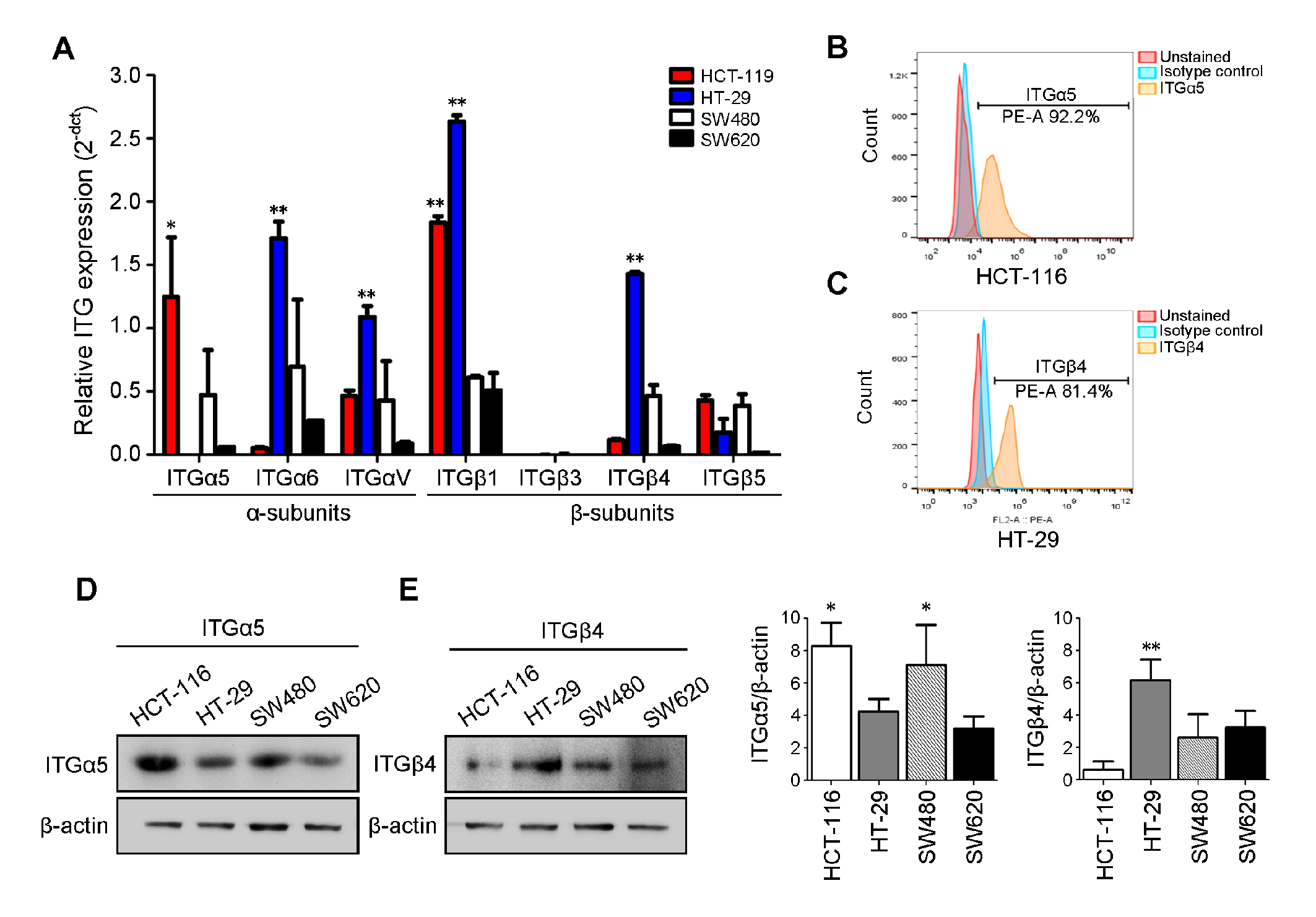

Supplement: Supplementary file 1 — Fig S1 [file JCMM-24-12421-s001.tif]

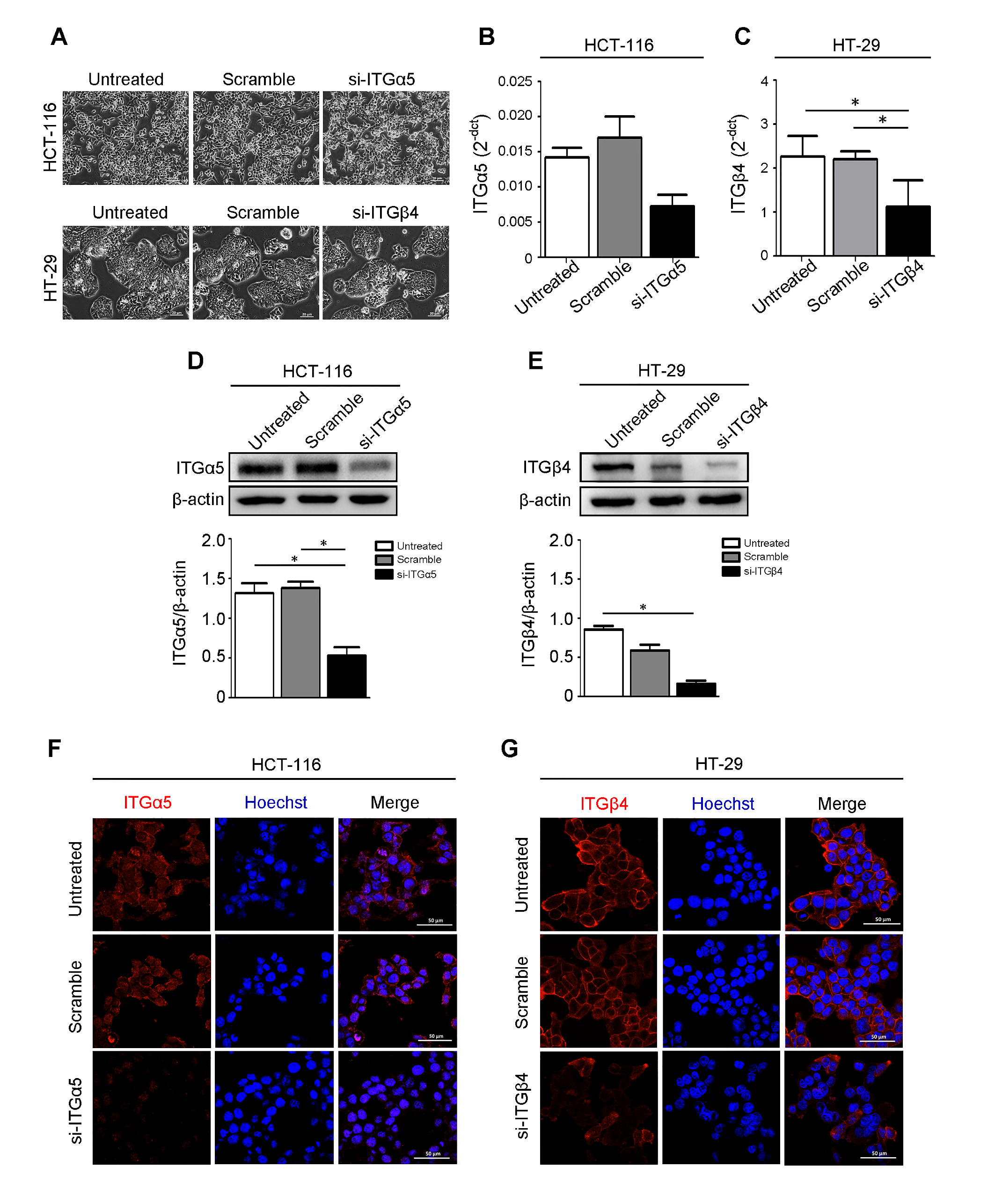

Supplement: Supplementary file 2 — Fig S2 [file JCMM-24-12421-s002.tif]

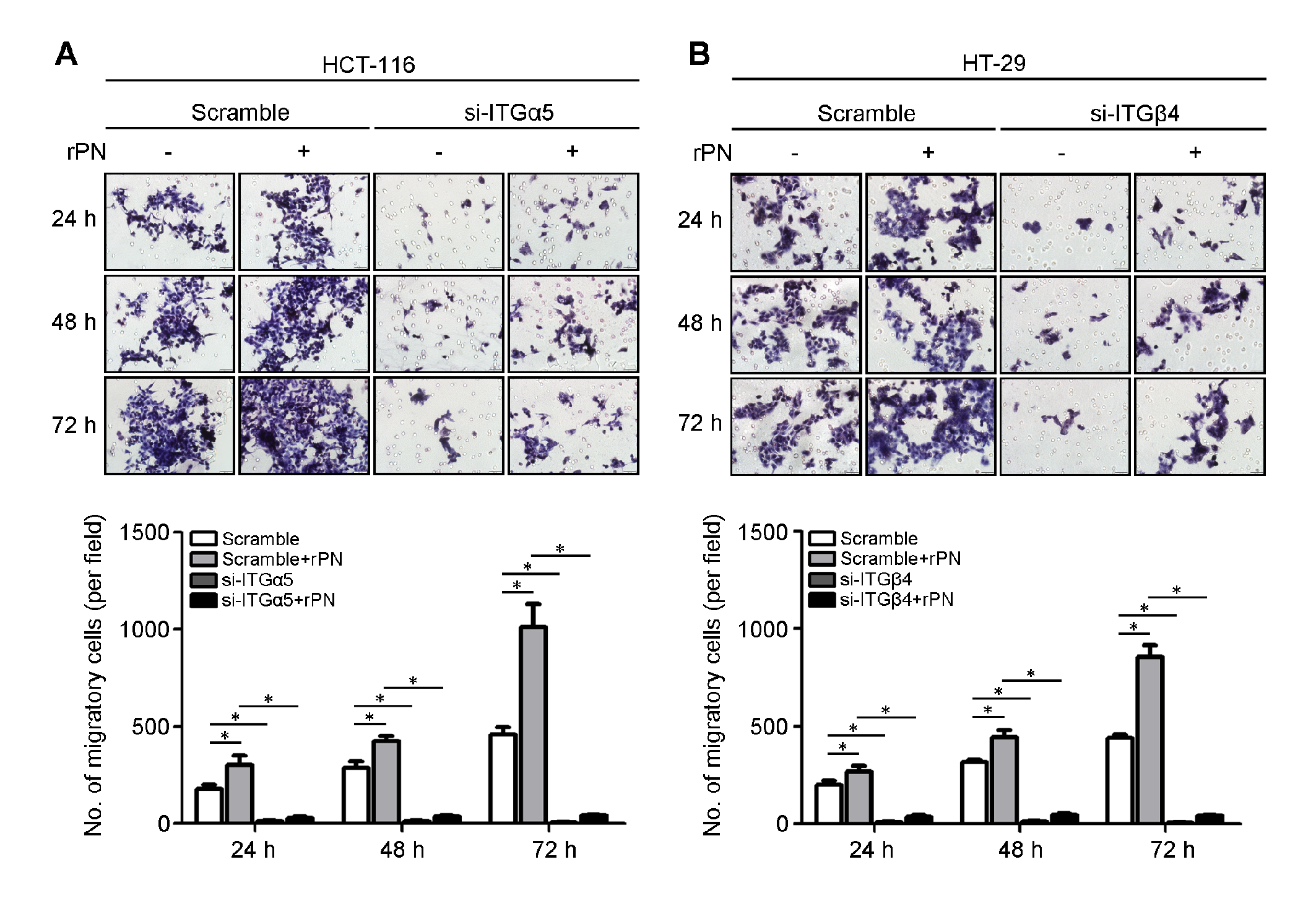

Supplement: Supplementary file 3 — Fig S3 [file JCMM-24-12421-s003.tif]
