# Supplementary material for: Periostin regulates autophagy through integrin α5β1 or α6β4 and an AKT‐dependent pathway in colorectal cancer cell migration
Source: J Cell Mol Med. 2020 Sep 29;24(21):12421–32. doi: 10.1111/jcmm.15756 (PMC7686974; doi:10.1111/jcmm.15756)
Supplement: Supplementary file 4 — Table S1 [file JCMM-24-12421-s004.docx]

**Supplement Table 1.** The correlation between tissue PN and serum CEA levels in CRC patients

|  |  | **CRC (n=351)** | | |
| --- | --- | --- | --- | --- |
|  |  | **Tissue periostin expression** | | |
|  |  | **Low (n=82)** | **High (n=269)** | **P-value** |
| **Serum CEA**  **(cut off 3.4 ng/ml)** | **Low (n =93)** | 29 | 64 | 0.0376* |
|  | **High (n =258)** | 53 | 205 |  |
